# Effect of electronic nicotine delivery systems for smoking cessation on sleep quality: secondary analysis of a randomized controlled trial

**DOI:** 10.1093/sleep/zsag028

**Published:** 2026-02-05

**Authors:** Tamara Scharf, Anna Rihs, Anna Schoeni, Micheline Maire, Kali Tal, Julian Jakob, Isabelle Jacot-Sadowski, Jean-Paul Humair, Aurélie Berthet, Martin Brutsche, Anja Frei, Lucy Bolt, Ramin Khatami, Reto Auer, Stéphanie Baggio

**Affiliations:** Institute of Primary Health Care (BIHAM), University of Bern, Bern, Switzerland; Graduate School of Health Sciences, University of Bern, Bern, Switzerland; Institute of Primary Health Care (BIHAM), University of Bern, Bern, Switzerland; Institute of Primary Health Care (BIHAM), University of Bern, Bern, Switzerland; Department of Internal Medicine, Stadtspital Zürich, Zürich, Switzerland; Institute of Primary Health Care (BIHAM), University of Bern, Bern, Switzerland; Institute of Primary Health Care (BIHAM), University of Bern, Bern, Switzerland; Graduate School of Health Sciences, University of Bern, Bern, Switzerland; Department of Paediatrics, University Hospital Bern, Inselspital, Bern, Switzerland; Center for Primary Care and Public Health (Unisanté), University of Lausanne, Lausanne, Switzerland; Department of Primary Care Medicine, University Hospital of Geneva, Geneva, Switzerland; Center for Primary Care and Public Health (Unisanté), University of Lausanne, Lausanne, Switzerland; Lung Center, Kantonsspital St. Gallen, St. Gallen, Switzerland; Epidemiology, Biostatistics and Prevention Institute, University of Zurich, Zurich, Switzerland; Institute of Primary Health Care (BIHAM), University of Bern, Bern, Switzerland; Department of Internal Medicine, University Hospital Bern, Inselspital, Bern, Switzerland; Centre of Sleep Medicine and Epileptology Barmelweid, Klinik Barmelweid AG, Barmelweid, Switzerland; Institute of Primary Health Care (BIHAM), University of Bern, Bern, Switzerland; Center for Primary Care and Public Health (Unisanté), University of Lausanne, Lausanne, Switzerland; Institute of Primary Health Care (BIHAM), University of Bern, Bern, Switzerland; Institute of Psychology, University of Lausanne, Lausanne, Switzerland

**Keywords:** E-cigarettes/electronic nicotine delivery systems (ENDS), tobacco smoking cessation, subjective sleep quality

## Abstract

**Study Objectives:**

E-cigarettes can help smokers quit, but how e-cigarettes used for tobacco smoking cessation impact sleep is still unclear. The primary objective was to evaluate the effect of e-cigarettes for smoking abstinence on sleep quality. Secondary objectives included subscales of sleep quality.

**Methods:**

We conducted a secondary analysis of the Efficacy, Safety, and Toxicology of electronic nicotine delivery systems for smoking cessation (ESTxENDS) randomized controlled trial, which included adult smokers in Switzerland (five sites, 7.2018-6.2021). The intervention group received free e-cigarettes and e-liquids over 6 months plus standard-of-care smoking-cessation counseling (SOC); the control group received SOC alone. The primary outcome was overall self-reported sleep quality at 6 months, measured by the Pittsburgh Sleep Quality Index (PSQI). We considered a minimal clinically important difference (MCID) of 2.5–5. Secondary outcomes included PSQI subscales. We used adjusted linear regressions with inverse probability of attrition weights (IPAW).

**Results:**

ESTxENDS included 1246 participants. Of these, 831 participants completed the PSQI at follow-up. For the primary outcome, there was no significant difference in PSQI score between groups (*b* = −0.20, *p* = .256, adjusted analyses with IPAW). For PSQI subscales, only sleep efficiency was significantly better in the intervention group (*b* = 1.87, *p* = .018), below MCID.

**Conclusion:**

E-cigarettes added to SOC for tobacco smoking abstinence did not significantly alter participant’s self-reported sleep quality compared to SOC alone. Clinicians can inform patients willing to quit smoking with e-cigarettes that their use is not likely to disrupt their perceived sleep quality on average.

## Graphical Abstract

**Graphical Abstract:**
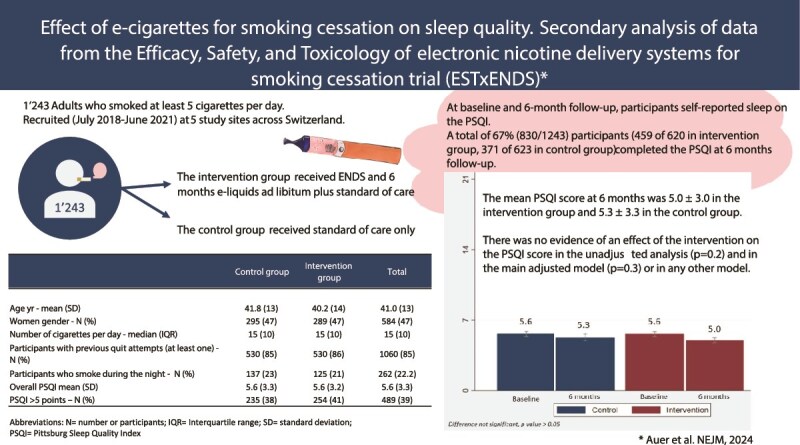

Statement of Significance While e-cigarettes are a proven effective method to increase the chances of abstaining from tobacco, clinicians and their patients need to know whether using e-cigarettes for smoking cessation affect sleep-related symptoms. This study provides the first evidence from a large randomized controlled trial that adding e-cigarettes to standard cessation counseling does not significantly alter perceived sleep quality over 6 months. In addition, e-cigarettes for smoking cessation may modestly improve sleep efficiency among individuals with poor baseline sleep quality. However, critical gaps remain regarding the short-term effects of nicotine withdrawal, the potential long-term impacts of sustained e-cigarette use, and objective sleep measures. Future research combining polysomnography or actigraphy with long-term detailed exposure assessments is needed to clarify the underlying mechanisms.

## Introduction

Cigarette smokers are 2.5 times more likely to suffer from poor sleep than non-smokers [1–3], and poor sleep is associated with worse health outcomes (e.g. obesity, diabetes, depression, anxiety, cardiovascular diseases, and increased mortality) [4, 5]. Both nicotine and combustion byproducts may contribute to sleep disruption. Exposure to secondhand smoke is associated with respiratory symptoms and disrupted sleep [6–10]. Nicotine, a psychostimulant, promotes wakefulness and increases arousal [11, 12]. But nicotine has a short half-life of 1–2 h and people who smoke tobacco cigarettes do not tend to have high nicotine blood levels during sleep [13–15]. However, people who quit smoking may experience nicotine withdrawal, which can disturb sleep in different ways. Patient reports and objective measures show them having a harder time falling asleep, sleeping for shorter periods of time, waking up more frequently during the night and taking longer to reach the rapid eye movement stage when they quit smoking, all contributing to worse subjective and objective sleep quality [11, 12]. These symptoms usually improve with sustained tobacco abstinence [12].

Determining the specific effects of tobacco smoke on sleep is challenging in the presence of nicotine, but research that separates the effects of nicotine from those of tobacco smoke may be easier with the advent of e-cigarettes or electronic nicotine delivery systems. E-cigarettes can deliver nicotine in concentrations comparable to traditional cigarettes. Inhaling nicotine through e-cigarettes shows a similar pharmacokinetic profile compared to tobacco smoking, including time to peak concentration and elimination. E-cigarettes deliver 9- to 450-fold lower levels of toxic compounds than conventional tobacco cigarettes [7], and typically do not release toxic tobacco-specific nitrosamines or polycyclic aromatic hydrocarbons [8]. However, e-cigarettes still release toxins such as formaldehyde when the e-liquid is heated [16, 17], which could disrupt sleep [18, 19]. E-cigarettes are opening a path for researchers to study the effects of nicotine on sleep, with lower confounding influence of additional toxins in tobacco cigarettes smoke.

Studies on e-cigarettes and their effect on sleep are scarce. Cross-sectional studies showed that e-cigarettes use was associated with worse sleep outcomes. Baiden et al. found adolescent e-cigarette users and dual users slept significantly less than adolescents who never used e-cigarettes or tobacco [20]. Kang et al. found that dual e-cigarettes and cigarette users had worse subjective sleep quality than cigarette-only smokers [21]. A study on subjective sleep quality and daytime symptoms in e-cigarette and cigarette dual users found that cigarette users slept less and e-cigarette use frequency predicted daytime sleepiness [22]. However, a causal effect of e-cigarettes on sleep can’t be inferred from cross-sectional studies since participants using e-cigarettes might differ from those not using e-cigarettes on key covariates also impacting sleep outcome. For example, health awareness, socioeconomic status, and pre-existing conditions, could predict the probability of both e-cigarettes use and sleep quality. E-cigarettes are also often used by people who smoke to help them quit. Therefore, sleep outcomes may be associated with past or recent tobacco use rather than e-cigarette use. We can test the effect of e-cigarettes for smoking abstinence on sleep quality by analyzing data from randomized controlled trials (RCTs) allocating a group to either use e-cigarettes or not and then comparing sleep outcomes of the two groups.

We thus conducted a secondary analysis of data collected in the Efficacy, Safety and Toxicology of Electronic Nicotine Delivery Systems (ESTxENDS) RCT [16]. We examined the effect of a smoking cessation intervention, which included free e-cigarettes and e-liquids over 6 months in addition to standard-of-care (SOC) counseling compared to SOC alone on self-reported sleep quality. The primary objective was to test the effect of the intervention on overall self-reported sleep quality, assessed using the Pittsburgh Sleep Quality Index (PSQI) total score at 6-month follow-up. Secondary objectives were (1) to test the effect of the intervention on PSQI subscale scores (subjective sleep quality, sleep onset latency, overall sleep duration, sleep efficiency [total hours asleep/total hours spent in bed], number of nightly awakenings, use of sleep medication, and daytime symptoms like sleepiness or lack of enthusiasm), and (2) to test the effect of the intervention on PSQI in the subgroup of participants with sleep problems (PSQI >5). We further conducted per-exposure analyses, exploring the associations between self-reported tobacco and/or e-cigarette use at 6-month follow-up and self-reported sleep quality at 6 months.

## Methods

### Study design and participants

This study was a pre-registered secondary analysis of the Efficacy, Safety and Toxicology of Electronic Nicotine Delivery Systems as an aid for smoking abstinence (ESTxENDS) trial (Trial Registration for the primary smoking abstinence outcome: NCT03589989, for the secondary sleep quality outcome: NCT03603353, published 27.07.2018, last update 13.11.2023) [16]. The local ethics committee of each participating study site approved the trial (reference number: 2017-02332).

The ESTxENDS trial was an open-label, multicenter, parallel arms, superiority RCT that tested the efficacy on smoking abstinence outcomes of a smoking abstinence intervention adding e-cigarettes to SOC compared to SOC alone. Participants were recruited via advertisements from July 2018 until June 2021 at five sites across Switzerland. ESTxENDS included adults smoking at least five cigarettes per day who were willing to quit smoking within the next 3 months. The trial excluded pregnant or breast-feeding women, persons who had used nicotine replacement therapy (NRT) or another smoking-abstinence drug in the previous 3 months, and persons who had regularly used e-cigarettes or tobacco-heating systems in the previous 3 months and those who could attend follow-up visits (full exclusion criteria are listed in the Supplementary Appendix of the main ESTxENDS publication) [16]. Persons who were interested in participating in the trial contacted trained study nurses at each local study site. Nurses asked participants to specify a target tobacco smoking quit date (TQD) and scheduled a baseline visit 1 week before this TQD. At the baseline visit, the nurses confirmed eligibility, collected written consent forms and collected a battery of questionnaires and clinical assessments. Participants were then randomized via an automated, centralized computer-based system (ratio 1:1) in a protected environment at the Clinical Trials Unit in Bern, Switzerland. Nurses and participants were aware of the group assignment. In this secondary analysis, we used data from the baseline visits which occurred 1 week before the TQD and the follow-up visit scheduled 6 months after TQD.

### Control group (SOC counseling only)

Study nurses provided SOC smoking abstinence counseling based on cognitive behavior therapy, motivational interviewing, and shared decision-making for smoking abstinence drug support, including NRT and smoking cessation recommendations adapted to nicotine dependence (see Supplementary Appendix of the main ESTxENDS publication) [17, 18]. Participants were counseled in-person at the baseline visit and by phone at TQD and Weeks 1, 2, 4, and 8 after TQD. Participants allocated to SOC received CHF 50 (50 USD) vouchers at the baseline visit, which they could use for any purpose.

### Intervention

In addition to SOC, the intervention group received two free e-cigarette devices at the baseline visit, where study nurses showed them how to use e-cigarettes, charge the device, fill it with e-liquid, and change the coil every 2 weeks. Participants from the intervention group could sample between 6 flavors and 4 nicotine concentrations (24 different e-liquids, in 19.6-, 11-, 6- and 0 mg/mL nicotine concentrations and 6 flavors: 2 tobacco, 1 menthol, 3 fruity), which were presented to them on an e-liquid testing board. Study nurses gave participants no more than 10 e-liquid bottles at the end of this baseline visit and advised them to use only the e-liquids we provided. Participants could use e-cigarettes ad libitum and re-order e-liquids whenever and in whatever amount they wanted, in whatever nicotine concentrations or flavors they preferred for 6 months (see Supplementary Appendix of the main ESTxENDS publication) [16]. A complete description of the intervention, comparator, and randomization procedures are available in the main ESTxENDS publication [16].

### Outcome measures

Study nurses asked participants about sleep symptoms using the Pittsburgh Sleep Quality Index questionnaire (PSQI) during in-persons visits [19]. The primary outcome was the PSQI overall score at 6-month follow-up. Secondary outcomes included subscales of the PSQI, measured at 6-month follow-up.

The PSQI is a validated questionnaire developed as a diagnostic tool by the University of Pittsburgh to assess changes in sleep quality in the previous month scored from 0 to 21, higher scores indicating worse sleep quality [19]. Subscales range from 0 to 3 points each and measure subjective sleep quality, sleep onset latency, overall sleep duration, sleep efficiency (total hours asleep/total hours spent in bed), number of nightly awakenings, use of sleep medication, and daytime symptoms like sleepiness or lack of enthusiasm.

We relied on the literature to set the cut-off for possible chronic insomnia on the overall PSQI score at 5 points and the minimal clinically important difference (MCID) at 2.5 to 5 points [23, 24].

### Other variables

#### Baseline characteristics related to tobacco smoking

Participants completed the Fagerström test for cigarette dependence (range 0–10). Higher scores indicated greater dependency. We counted the number of cigarettes smoked daily, the number of previous quit attempts, and whether participants smoked during the night or used other tobacco products in the 6 months before their baseline visit.

#### Tobacco and e-cigarette exposure

For the per-exposure analysis, we classed participants of the intervention group into five groups based on their self-reported exposure to tobacco cigarettes and e-cigarettes in the 7 days prior to the 6-months follow-up visit and/ or use of NRT within the last 24 h, as done previously: “tobacco and e-cigarette abstainers” reported no use of tobacco cigarettes or e-cigarettes; “e-cigarettes with nicotine users” reported no use of tobacco cigarettes, e-cigarettes with nicotine, or NRT; “e-cigarette without nicotine users” reported no use of tobacco cigarettes but the use of e-cigarettes without nicotine; “dual users” reported the use of both tobacco cigarettes and e-cigarettes (with or without nicotine); and “tobacco smokers” reported the use of tobacco cigarettes but not e-cigarettes. Because the nature of e-cigarette exposure differed between groups—encouraged and structured in the intervention arm versus self-initiated in the control—we limited our exposure-based analyses to the intervention group. ESTxENDS assessed a range of smoking cessation outcomes at 6-month follow-up. We used the 7-day point-prevalence abstinence from smoking (7-dppa) for primary analyses, which we computed based on self-reported use of any tobacco cigarettes or e-cigarettes, with or without nicotine [25]. We further defined sustained abstinence at 6 month follow up as no smoking with a 2-week grace period after the TQD. In the analyses, we distinguished between “sustained tobacco and e-cigarette abstainers” and “7-day tobacco and e-cigarette abstainers”. We defined biochemically validated abstinence with urinary anabasine levels (<3 ng/mL in urine), or if unavailable, exhaled carbon monoxide level of ≤9 ppm. In the analyses, we considered “tobacco abstainers” (with or without concomitant e-cigarette use) as being abstainers if abstinence was biochemically validated. Participants reporting self-reported abstinence without validation were considered as “tobacco smokers.” For participants using e-cigarettes, we recorded nicotine concentration for all e-liquids reported. If participants used a variety of nicotine concentrations in the same week, we calculated mean nicotine concentration by summing up the nicotine concentrations and dividing them by the number of different concentrations. We assessed exposure to nicotine-replacement therapy within the 24 h before the 6-month visit.

#### Sociodemographic and health variables

We collected data on gender, weight, height, education level, and working situation. We asked if participants had been diagnosed with obstructive sleep apnea syndrome, which influences sleep quality, and if they had been or were being treated for this disease. Participants completed the Alcohol Use Disorders Identification Test-Concise (AUDIT-C) to estimate the frequency and quantity of alcohol consumption and the number of times a year participants consume six or more standard drinks. We used the usual AUDIT-C cut-off score of ≥3 points for women or ≥4 points for men to determine if participants had potentially problematic alcohol use [26]. Participants were asked if they had used cannabis at least once in their lifetime. We used the Patient Health Questionnaire-9 items (PHQ-9) at baseline to assess self-reported depressive symptoms and their severity. The PHQ-9 is a validated questionnaire based on the DSM-IV’s diagnostic criteria for major depression; PHQ-9 also aligns with DSM-5’s new measures of depression severity. Total score indicates the severity of suspected depression (range: 0–27). We assessed symptoms of generalized anxiety disorder (GAD) at baseline with the validated GAD-7 item questionnaire. This tool, validated against the DSM-IV’s diagnostic criteria for GAD, is also recommended for evaluating the severity of DSM-5 GAD [27]. Total score indicates severity of anxiety symptoms (range: 0–21). Patients provided details of their current medication. In this study, we reported those that could influence sleep: antipsychotics, antidepressants, hypnotics, and sedatives, melatonin receptor agonists, central sympathomimetics, and anxiolytics. They were recorded as present or absent at baseline and we created a single binary category of medication influencing sleep. Patients self-reported recreational drug use (amphetamines, psilocybin, LSD, ecstasy, phencyclidine, cocaine, heroin, morphine, methadone, and codeine). As done in a previous study [28], we assessed presence of any of: problematic alcohol use at baseline, defined as an AUDIT-C) [26] score of ≥4 points for men and ≥3 points for women; problematic cannabis use during the 6 months before baseline, defined as a score ≥8 on the Cannabis Use Disorder Identification Test-Revised [29]; and polysubstance use during the 6 months before baseline, defined as use of at least two illicit substances. A binary variable of problematic substance use was created.

### Statistical analysis

We did not compute sample size for this secondary analysis since sample size had been computed for the primary ESTxENDS outcome (biochemically validated continuous tobacco smoking abstinence at 6-month follow-up) [16]. The protocol for the main trial as well as the statistical analysis plan was published together with the main paper as an Appendix.

We first summarized baseline characteristics with descriptive statistics. We explored patterns of missing values and used simple imputation to fill in missing baseline values. We imputed 53 values of baseline characteristics for participants who had completed the PSQI at 6 months: 3 values for the body mass index (BMI); 32 for the baseline PSQI; 7 for the AUDIT-C score; 2 for the baseline PHQ-9; 3 for the baseline GAD-7, 2 for ever consuming cannabis, 2 for the Fagerström Score, 2 for if the participants used other tobacco products in the 6 months before their baseline visit, and 2 for problematic substance use. We considered participants who reported use of e-cigarettes at 6-months with missing data on nicotine concentration in the e-liquid they used as using e-cigarettes without nicotine, as done previously [16]. For the subscales of PSQI, two outliers with implausible values were replaced by the next highest value.

We conducted main analyses using linear regression models to compare groups (intervention vs. control) based on their primary (overall sleep quality) and secondary (subscores of sleep quality) outcomes, including all participants in their original randomization group. The subscales sleep disturbance, dysfunction during the day, sleep medication, and sleep quality were analyzed using ordered logistic regression. We used the same method to compare sleep efficiency subscores between groups. We performed ad hoc subgroup analyses on participants with a PSQI score >5 at baseline [23].

For the per-exposure analysis, we used linear regression models to test the association between the exposure groups and performed a range of exploratory analyses with alternate exposure group categories, using an additional category for sustained abstinence and using validated abstinence.

To address potential selection bias caused by attrition, we applied inverse probability of attrition weights (IPAW), which recent RCTs used successfully [30, 31]. This resulted in adjusted models, which controlled for baseline variables selected using directed acyclic graphs (baseline levels of sleep quality, smoking characteristics, problematic substance use, sociodemographic, and health variables), as well as anxiety and depression scores, and cigarette dependance scores. This set of variables are theoretically related to the risk of attrition. We also assumed the missing data was missing at random. We did not impute data at 6 months. We present unadjusted and adjusted models. As adjusted models were more precise and had more power, we considered those as our primary analyses. Stata software, version 17 (StataCorp) was used for all analyses, subgroup and subscale analyses.

## Results

### Baseline characteristics

We included 1246 participants (intervention group 622, control group 624); median age was 38 years (Interquartile range: 29–51); 47 per cent were women (Table 1); 1179 (95 per cent) completed the PSQI at baseline. Mean PSQI scores at baseline were 5.6 in the intervention group and 5.6 in the control group. At baseline, 41 per cent participants in the intervention and 37.7 per cent in the control group achieved a PSQI score of >5 points.

**Table 1:** Characteristics of the participants at baseline

	**Control group**	**Intervention group**	**Total**
	***N* = 624**	***N* = 622**	***N* = 1246**
**Age in years—median (IQR)**	39 (30–52)	37 (28–51)	38 (29–51)
**Women gender—*N* (%)**	295 (47.3)	290 (46.6)	585 (47.0)
**Employed—*N* (%)**	465 (74.5)	438 (70.4)	903 (72.5)
**BMI—mean (SD)**	25.7 (4.9)	25.6 (4.9)	25.6 (4.9)
**Highest educational qualification—*N* (%)**			
Obligatory school; other; none	45 (7.2)	50 (8.0)	95 (7.6)
Secondary education	277 (44.4)	291 (46.8)	568 (45.6)
Tertiary education	302 (48.4)	281 (45.2)	583 (46.8)
**Smoking behavior**			
Age started smoking in years**—**median (IQR)	16 (15–19)	16 (15–18)	16 (15–19)
Number of cigarettes per day**—**median (IQR)	15 (10–20)	15 (10–20)	15 (10–20)
Participants with previous quit attempts (at least one)**—***N* (%)	530 (84.9)	531 (85.4)	1061 (85.2)
Participants who smoke during the night**—***N* (%)	146 (23.4)	134 (21.5)	280 (22.5)
Fagerström test for nicotine dependence**—**mean (SD)^*^	4.4 (2.3)	4.3 (2.3)	4.3 (2.3)
**Medication influencing sleep**			
Antipsychotic medication	22 (3.5)	27 (4.3)	49 (3.9)
Antidepressants	79 (12.7)	72 (11.6)	151 (12.1)
Hypnotics and sedatives	20 (3.2)	18 (2.9)	38 (3.1)
Melatonin receptor agonists	6 (1.0)	2 (0.3)	8 (0.6)
Central sympathomimetics	13 (2.1)	15 (2.4)	28 (2.3)
Anxiolytic benzodiazepines	19 (3.1)	23 (3.7)	42 (3.4)
Any medication influencing sleep	110 (17.6)	107 (17.2)	217 (17.4)
**Mental health variables and further substance use**			
PHQ-9 score**—**median (IQR)	3 (1–6)	3 (1–6)	3 (1–6)
GAD-7 score**—**median (IQR)	4 (2–7)	5 (2–8)	4 (2–8)
Participants with OSAS diagnosis**—***N* (%)	19 (3.0)	29 (4.7)	48 (3.9)
Alcohol use**—**AUDIT-C score ≥3 women/≥4 men - *N* (%)	393 (63.0)	366 (58.8)	759 (60.9)
Cannabis use at least once in lifetime**—***N* (%)	524 (84.0)	541 (87.0)	1065 (85.5)
Problematic substance use**—***N* (%)^†^	119 (19.7)	113 (18.2)	232 (18.6)
**PSQI at baseline** ^‡^ **—mean (SD)**	5.6 (3.3)	5.6 (3.2)	5.6 (3.3)
Sleep duration in hours (mean, SD)	6.7 (1.2)	6.8 (1.1)	6.8 (1.2)
Sleep disturbance: Never—*N* (%)	24 (3.9)	26 (4.2)	50 (4.0)
Less than once a week—*N* (%)	451 (72.3)	465 (74.8)	916 (73.5)
Once to three times a week—*N* (%)	125 (20.0)	114 (18.3)	239 (19.2)
Sleep latency in minutes—median (IQR)	15 (10–30)	15 (10–30)	15 (10–30)
Dysfunction during the day: Never—*N* (%)	175 (28.0)	172 (27.7)	347 (27.9)
Less than once a week—*N* (%)	351 (56.3)	355 (57.1)	706 (56.7)
Once to three times a week**—***N* (%)	90 (14.4)	92 (14.8)	182 (14.6)
Sleep efficiency in percent**—**mean (SD)	88.7 (12.8)	87.9 (11.9)	88.3 (12.4)
Sleep quality: Very good**—**rather good**—***N*(%)	479 (76.8)	468(75.2)	947 (76.1)
Rather bad**—**very bad**—***N*(%)	142 (22.8)	151 (24.3)	293 (23.5)
Medication for sleep: Never**—***N* (%)	517 (82.9)	517(83.1)	1034 (83.0)
Less than once a week**—***N* (%)	38 (6.1)	37 (6.0)	75 (6.0)
Once to three times a week**—***N* (%)	65 (10.4)	65 (10.5)	130 (10.4)
PSQI >5 points**—***N* (%)	235 (37.7)	256 (41)	491 (39.4)

^*^Scores range from 1 to 10, with higher scores indicating greater dependence.

^†^Any of the following: problematic alcohol use at baseline, defined as an AUDIT-C score of ≥4 points for men and ≥3 points for women; problematic cannabis use during the 6 months before baseline, defined as a score ≥8 on the CUDIT-R; and polysubstance use during the 6 months before baseline, defined as use of at least two illicit substances.

^‡^Score ranges from 1 to 21, higher scores indicate worse sleep quality.

AUDIT-C, Alcohol Use Disorders Identification Test-Concise; BMI, body mass index; CUDIT-R, Cannabis Use Disorder Identification Test-Revised; GAD-7, Generalized Anxiety Disorder-7 items; IQR, interquartile range; *N*, number or participants; OSAS, obstructive sleep apnea syndrome; PHQ-9, Patient Health Questionnaire-9 items; PSQI, Pittsburgh Sleep Quality Index; SD, standard deviation.

### Exposure to tobacco smoking and e-cigarettes with nicotine at 6-month follow-up

Data on tobacco and e-cigarette use was available for 827 (66 per cent) participants, who also provided data on the PSQI at 6-month follow-up (*n* = 371 in the control group; *n* = 456 in the intervention group). At 6-month follow-up, 41.8 per cent (155/371) in the control group and 62.5 per cent (285/456) in the intervention group reported abstaining from smoking cigarettes in the 7 days prior to the assessment. A total of 243 participants reported using exclusively e-cigarette, 234 (96 per cent) in the intervention group and 9 (4 per cent) in the control group. A total of 193 (79 per cent) participants (all in the intervention group) reported using e-cigarettes with nicotine. Of the 93 participants reporting dual use, 80 (86 per cent) were in the intervention group and 13 (12 per cent) were in the control group.

### Main analyses

**Figure 1:**
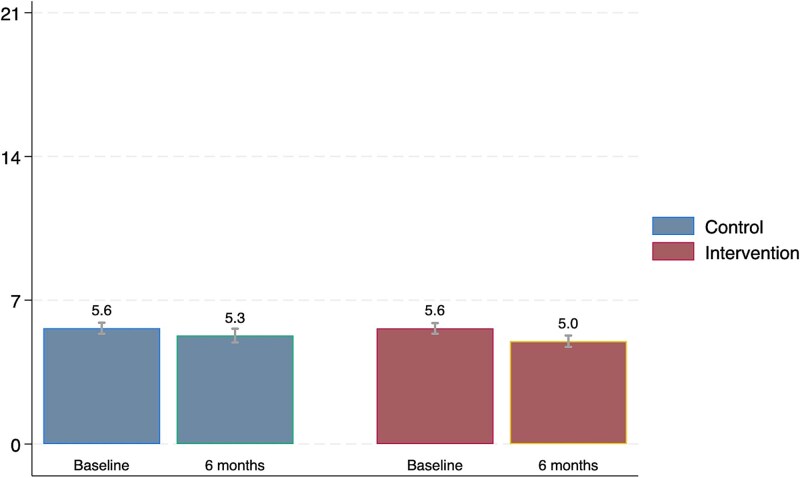
Mean Pittsburgh Sleep Quality Index scores.

A total of 831 participants (67 per cent), 460 (74.0 per cent) in the intervention group, and 371 (59.5 per cent) in the control group, completed the PSQI at 6-month follow-up. Mean PSQI score at 6 months was 5.0 in the intervention group and 5.3 in the control group (Table 2, Figure 1). We found no evidence that the intervention influenced PSQI scores in our unadjusted analysis. The difference between means was −0.28 (95% confidence interval [CI] = −0.71 to 0.15, *p* = .205), which was below the MCID we set. Similarly, the main adjusted model showed no significant effect (*p* = .256; see Table 3). From baseline to the 6-month follow-up, the mean PSQI score decreased was −0.35 ± 0.13 in the intervention group and −0.2 ± 0.15 in the control group. The slope of the difference was −0.20 (95% CI = −0.55 to 0.15, *p* = .256) in the adjusted model.

**Table 2:** Descriptive statistics for sleep quality at 6 months

**Outcome**	**Control group** ***N* = 371**	**Intervention group** ***N* = 460**	**Total** ***N* = 831**
**PSQI score—mean (SD)**	5.3 (3.3)	5.0 (3.0)	5.1 (3.1)
**Change in PSQI score from BL to M6—mean (SE, 95% CI)**	−0.18 (0.15, −0.46 to 0.11)	−0.33 (0.13, −0.59 to −0.08)	−0.26 (0.10, −0.45 to −0.07)
**PSQI subscales**			
Sleep duration in hours**—**mean (SD)	6.9 (1.2)	6.9 (1.1)	6.9 (1.2)
Sleep disturbance:			
Never**—***N* (%)	25 (6.7)	33 (7.2)	58 (7)
Less than once a week**—***N* (%)	282 (76.0)	363 (78.9)	645 (77.6)
Once to three times a week**—***N* (%)	64 (17.3)	64 (13.9)	128 (15.4)
Sleep latency in minutes**—**median (IQR)	15 (7–30)	15 (10–30)	15 (10–30)
Dysfunction during the day:			
Never**—***N* (%)	124 (33.4)	142 (30.9)	266 (32.0)
Less than once a week**—***N* (%)	195 (52.6)	261 (56.7)	456 (54.9)
Once to three times a week**—***N* (%)	52 (14.0)	57 (12.4)	109 (13.1)
Sleep efficiency in percent**—**mean (SD)	87.8 (12.5)	89.7 (10.5)	88.9 (11.5)
Sleep quality:			
Very good/rather good**—***N*(%)	295 (79.5)	379 (82.4)	674 (81.1)
Rather poor/very poor**—***N*(%)	76 (20.5)	81 (17.6)	157 (18.9)
Medication for sleep:			
Never**—***N* (%)	317 (85.4)	394 (85.7)	711 (85.6)
Less than once a week**—***N* (%)	17 (4.6)	19 (4.1)	36 (4.3)
Once to three times a week**—***N* (%)	37 (10.0)	47 (10.2)	84 (10.1)

6M, 6-month follow-up; BL, baseline; IQR, interquartile range; *N*, number or participants; PSQI, Pittsburgh Sleep Quality Index; SD, standard deviation.

**Table 3:** Unadjusted and adjusted models for sleep quality at 6 months comparing intervention and control groups

	**Unadjusted**		**Final model with SI/IPAW** ^*^		
**Outcome**	**Slope (95% CI)**	***p*-value**	**Slope (95% CI)**	***p*-value**	***R*** ^**2**^
**PSQI score**	−0.28 (−0.71 to 0.15)	.205	−0.20 (−0.55 to 0.15)	.256	0.40
**Change in PSQI score**	−0.16 (−0.54 to 0.23)	.421	−0.20 (−0.55 to 0.15)	.256	0.25
Sleep duration	0.06 (−0.09 to 0.21)	.438	0.09 (−0.05 to 0.24)	.219	0.11
Sleep disturbance	−0.18 (−0.50 to 0.14)	.274	−0.14 (−0.48 to 0.20)	.422	0.12†
Sleep latency	0.82 (−1.94 to 3.58)	.561	0.97 (−1.74 to 3.67)	.483	0.17
Dysfunction during the day	0.04 (−0.22 to −0.30)	.761	0.06 (−0.21 to 0.34)	.662	0.11†
Sleep efficiency	1.83 (0.24 to 3.42)	.024	1.87 (0.32 to 3.42)	**.018**	0.15
Sleep quality	−0.13 (−0.39 to 0.12)	.304	−0.14 (−0.40 to 0.14)	.330	0.15†
Medication for sleep	−0.04 (−0.40 to 0.32)	.834	0.01 (−0.41 to 0.42)	.946	0.15^†^
Change in sleep efficiency BL to M6	2.53 (0.74 to 4.32)	.006	2.77 (0.97 to 4.57)	**.003**	0.12

^*^Multivariable adjusted model, adjusted for study site, age, gender, employment status, body mass index, education, problematic substance use, use of medication influencing sleep, Patient Health Questionnaire-9 items score, Generalized Anxiety Disorder-7 items score, baseline PSQI score, number of cigarettes per day, and Fagerström score, the model was stabilized with IPAW.

^†^Pseudo *R*^2^ are reported, as outcomes were ordinal variables.

BL, baseline; CI, confidence interval; IPAW, inverse probability of attrition weights; M6, 6-month follow-up; PSQI, Pittsburgh Sleep Quality Index; SI, simple imputation.

When examining PSQI questionnaire subscales, we found that sleep efficiency increased in the intervention group from 88.1% to 89.7% and decreased in the control group from 88.7% to 87.8% (adjusted model, slope 1.87, 95% CI = 0.32 to 3.42. *p* = .018) (see Tables 2 and 3). Otherwise, we found no significant effect of the intervention on other PSQI components (*p* ≥ .219) (Table 3).

When we restricted our analysis to individuals at risk for insomnia (PSQI >5), baseline PSQI mean score was 8.9 in the control group and 8.6 in the intervention group. At follow-up, we found PSQI had dropped to 6.7 in the intervention group and 7.6 in the control group. The slope was −0.68 (95% CI = −1.30 to −0.06, *p* = .031) in the adjusted model. Sleep efficiency significantly increased in the intervention group. At 6 months, average sleep efficiency in the intervention group was 87.7 per cent while in the control group it was 82.1 per cent; difference in sleep efficiency was 5.10 (95% CI = 2.29 to 7.91, *p* < .001). The slope of the change was 5.70 (95% CI = 2.44 to 8.96, *p* = .001). There was also a significant difference for sleep duration, with a longer sleep duration in the intervention group (slope = 0.31, 95% CI = 0.05 to 0.58, *p* = .021). Other subscales revealed no difference between control and intervention group at 6-month follow-up, except for dysfunction during the day, which was significantly lower in the intervention group in the unadjusted model (Supplementary Appendix Table 1, Supplementary Appendix Figures 1 and 2).

### Analyses on the association between e-cigarettes and/or tobacco exposure at 6-months follow-up and PSQI

Of the 460 participants in the intervention group who completed the PSQI at 6-month follow-up, 4 gave no information on their exposure; 11 per cent (51/456) of participants were classified as “tobacco and e-cigarettes abstainers”; 42 per cent (193/456) as “e-cigarettes with nicotine users”, 9 per cent (41/456) as “e-cigarettes without nicotine users”; 20 per cent (91/456) as “dual users” and 18 per cent (80/456) as “tobacco users” (Table 4). Baseline characteristics according to these categories of exposure are reported in Supplementary Appendix Table 2.

**Table 4:** Per-exposure analysis of the intervention group only comparing the sleep quality at 6 months

**Groups**	***N* (%)**	**Mean PSQI score at 6 months (SD)**	**Unadjusted**		**Final model with SI/IPAW** ^*^	
			**Slope (95%CI)**	***p*-value**	**Slope (95%CI)**	***p*-value**
**Tobacco and e-cigarette abstainers**	51 (11%)	5.3 (3.6)	Ref.	Ref.	Ref.	Ref.
**e-cigarettes with nicotine**	193 (42%)	4.9 (2.9)	−0.48 (−1.42 to 0.46)	.318	−0.94 (−1.82 to −0.05)	**.038**
**e-cigarettes without nicotine**	41 (9%)	5.3 (2.9)	−0.02 (−1.27 to 1.24)	.980	−0.42 (−1.57 to 0.73)	.476
**Dual users**	80 (18%)	5.3 (3.3)	−0.08 (−1.15 to 0.99)	.878	−0.55 (−1.64 to 0.55)	.328
**Exclusive smokers**	91 (20%)	4.7 (2.7)	−0.61 (−1.65 to 0.44)	.253	−0.73 (−1.73 to 0.27)	.151
					***R*** ^**2**^ **= 0.40**	

Ref., reference; CI, confidence interval; IPAW, inverse probability of attrition weights; PSQI, Pittsburgh Sleep Quality Index; SD, standard deviation; SI, simple imputation.

^*^Multivariable adjusted model, adjusted for study site, age, gender, employment status, body mass index, education, problematic substance use, use of medication influencing sleep, Patient Health Questionnaire-9 items score, Generalized Anxiety Disorder-7 items score, baseline PSQI score, number of cigarettes per day, and Fagerström score, the model was stabilized with IPAW.

Table 4 reports the results for mean PSQI score at 6-month follow-up. The linear regression that used tobacco and e-cigarette abstainers as the reference group did not show statistically significant differences in PSQI score in e-cigarettes with nicotine users in the main adjusted model; the slope was −0.94 (95% CI = −1.82 to −0.05, *p* = .038) (Table 4).

When we analyzed individual subscales of the PSQI questionnaire, we found sleep disturbance decreased more among those who used e-cigarettes with nicotine than among tobacco and e-cigarette abstainers (slope −1.50, *p* = .002). When we compared tobacco and e-cigarette abstainers to e-cigarettes without nicotine users, dual users, and tobacco users, we found no significant difference in sleep onset latency, sleep duration, time awake after sleep onset, day symptoms, subjective sleep quality, sleep efficiency, or use of sleep medication (*p* ≥ .086) (Supplementary Appendix Tables 3 and 4). When we restricted our analysis only to participants at high risk for insomnia (PSQI >5) and compared them to tobacco and e-cigarette abstainers, we found no significant difference in PSQI for the exposure groups (*p* ≥ .153) (Supplementary Appendix Table 5).

Of the reported e-cigarette and dual users in the intervention group we obtained information on nicotine concentration of 344 participants. A total of 37 used no nicotine and 301 used a variety of nicotine levels between 1 and 20 mg/mL. Mean nicotine concentration level was 6.8 (SD 4.4). We found no significant relation between PSQI and nicotine levels in our unadjusted and adjusted models (slope 0.09, 95% CI = −0.02; 0.19 *p* = .07 unadjusted; slope 0.05, 95% CI = −0.04 to 0.14, *p* = .262 adjusted).

We further contrasted participants based on alternate definitions of tobacco/e-cigarette exposure. Out of 460 participants, 3 per cent (13/460) were classed into “sustained tobacco abstainers, e-cigarette abstainers”, 8 per cent (38/460) into “7 day tobacco abstainers, e-cigarette abstainers”, 42 per cent (193/460) into “e-cigarettes with nicotine”, 9 per cent (41/460) into “e-cigarettes without nicotine”, 18 per cent (80/460) into “dual users,” and 20 per cent (91/460) “tobacco smokers” (Supplementary Appendix Table 6). The linear regression that used “sustained tobacco abstainers, e-cigarette abstainers” as the reference group did not show statistically significant differences in PSQI score across categories (*p* > .099) (Supplementary Appendix Table 6). We further examined the validated tobacco and e-cigarette abstainers (10 per cent; 44/460) comparing them to e-cigarette with and without nicotine users, dual users and exclusive smokers, the linear regression demonstrated a significant difference in PSQI scores between the validated tobacco and e-cigarette abstainers and the e-cigarette with nicotine users; the slope was −1.19 (95% CI = −2.15 to −0.23, *p* = .015) (Supplementary Appendix Table 7).

## Discussion

A smoking cessation intervention in which participants received free e-cigarettes containing nicotine for 6 months added to SOC counseling did not significantly alter participant’s self-reported quality of sleep on average compared to participants who received SOC counseling alone. The intervention did not significantly lead to differences in sleep onset latency, overall sleep duration, use of sleep medication, or daytime symptoms. When we analyzed PSQI subscales, we found the intervention significantly increased sleep efficiency, though the change was not of clinical importance. In our secondary per exposure analysis, the change in PSQI score did not significantly differ between those who used e-cigarettes with nicotine compared to those who abstained from tobacco and e-cigarettes. Among participants with PSQI score > 5, overall PSQI scores improved more in the intervention group than in the control group with a mean difference of 1.9 compared to 1.3 in the control group. This was below the MCID of 2.5–5 [24].

Our medium-term (6 months) study of the effects of smoking cessation interventions on sleep quality provides insights that align with existing research. Although we can only test the effect of the intervention at 6-month follow-up and not in between, our results align with those of a literature review of clinical studies and self-reports, which found that sleep quality initially decreased after smoking cessation [32] but improved again within 3 to 12 months [12, 33]. Our intervention group, which received e-cigarettes and mostly used nicotine-containing e-liquids, did not experience significant changes in overall sleep quality, sleep onset latency, or sleep duration compared to the control group. Results were robust when testing the association between self-reported nicotine concentration used in e-cigarettes and sleep outcome. This aligns with previous clinical studies indicating that nicotine’s short half-life of 1–2 h may limit its sleep-disrupting effects, especially later during the night [11, 34]. The improvement in sleep efficiency observed in our intervention group, although not of clinical importance, suggests that switching from tobacco cigarettes to nicotine-containing e-cigarettes may have some positive effects on sleep. This supports the hypothesis that tobacco smoke, and not nicotine, impairs sleep among tobacco smokers, possibly through respiratory irritation [35–37].

Our findings support earlier evidence from a US survey suggesting e-cigarettes users was not associated with lower sleep quality (mean PSQI 6.87, SD 3.65) than non-smokers (mean PSQI 7.09, SD 3.41) [38]. However, our results are not in line with cross-sectional survey studies that linked e-cigarettes use to insufficient sleep [20, 39]. Results also do not support Merianos et al. which reported that adolescents who exclusively used e-cigarettes had more insufficient sleep than cigarette users, with dual users experiencing the highest rates of insufficient sleep [39]. These conflicting results may stem from differences in study designs, populations, and methods. Unlike cross-sectional studies that can only establish associations, our randomized trial allows for stronger inferences about causality for smokers willing to quit smoking with or without e-cigarettes. While many surveys focus on adolescents or the general population, our study specifically examines adult smokers attempting to quit—a clinically relevant group with different nicotine exposure histories and motivations for use. By providing e-cigarettes as part of a structured smoking cessation intervention, we also ensure more standardized exposure, reducing variability seen in self-reported use. Moreover, while studies on adolescents often frame e-cigarette use as a potential gateway to nicotine addiction and even subsequent tobacco smoking, our study investigates their role as a cessation aid. These differences highlight the need for more longitudinal studies and randomized controlled trials to further clarify the relationship between e-cigarettes, smoking cessation, and sleep quality.

### Limitations

Our study had several limitations. First, while the PSQI gave us valuable insight into participants’ sleep patterns, attrition rate was high at 6 months (34 per cent for the PSQI, 40.5 per cent in the control group and 26.0 per cent in the intervention group). ESTxENDS was designed to assess the effect of the intervention on smoking cessation outcomes and whenever patients did not come for clinical visits, we prioritized assessments of the primary and smoking cessation-related outcomes and not the secondary outcomes. We took attrition into account using IPAW to minimize its effect. IPAW uses weights to balance groups of participants based on their probability of staying in the study. This method ensures that the final analysis more accurately reflects the characteristics of the full original sample, including those who dropped out, under the assumption of missing at random. Second, we operated within the scope of the larger ESTxENDS trial, so our power and sample size calculations were based on the trial aims of smoking cessation and not sleep quality; this may have decreased our power to identify significant results in our study. Even with pre-registration with a separate entry in Clinicaltrial.gov for these secondary analyses, we consider these analyses as secondary and encourage readers to do so. Third, PSQI were collected at only two time points (baseline and 6-month follow-up visit); the calculated change score does not imply a linear progression, it is possible that during the 6 months there were other oscillations of the sleep quality. Fourth, psychiatric comorbidities were assessed using self-administered screening tools (PHQ-9 and GAD-7) rather than formal clinical evaluations, which may limit diagnostic accuracy. Participants also reported their use of antidepressant and anxiolytic medication at baseline, offering further insight into psychiatric conditions requiring treatment. However, this information depends on participant disclosure and reflects only those under pharmacological care at study entry. Fifth, we did not report attrition rates stratified by exposure group, which limits our ability to assess potential differential dropout. If participants with certain nicotine exposure profiles were more likely to be lost to follow-up, this could introduce bias and affect the validity of per-exposure analyses. While we attempted to account for this with IPAW, incomplete information on attrition by exposure may still limit the accuracy of these analyses. Sixth, during the trial, about 5 per cent of participants in the control group used e-cigarettes purchased outside of the trial. We expect the use of e-cigarettes in the control group to drive the null hypothesis of no difference between groups on sleep outcomes. Seventh, ESTxENDS tested the comparative effectiveness of providing e-cigarettes added to SOC to SOC alone and the participants were not blinded to the intervention. ESTxENDS did not test the differential effect of nicotine-containing e-cigarettes vs non-nicotine e-cigarettes (which could serve as a placebo intervention). Our analyses cannot tease out the specific effect of nicotine in e-cigarettes on our tested outcomes. Lastly, our data relied on self-reported sleep quality using the PSQI, without objective validation through actigraphy or polysomnography, introducing potential biases, such as recall bias or individual differences in sleep perception, which could affect the reliability of our findings [40]. However, since sleep quality is inherently subjective and strongly linked to an individual’s experience of restfulness and daytime functioning, self-reported data like ours remain relevant [41].

Since our study was based on data from one smoking cessation trial conducted in Switzerland (the ESTxENDS trial) generalization of our results is limited to tobacco smokers in Switzerland, who consume five or more cigarettes per day, and who are willing to abstain from tobacco smoking.

### Implications

Future research should integrate objective measures such as polysomnography and actigraphy to determine the effects of e-cigarettes and short-term nicotine withdrawal on sleep quality. Smoking cessation interventions that include e-cigarettes do not appear to degrade subjective sleep quality over the medium term (6 months) and may even benefit individuals at higher risk of insomnia. Our results add to the evidence that combining e-cigarettes with SOC counseling is a viable and sleep-neutral option for smoking cessation. However, it’s crucial to balance this potential benefit against the risk of developing a new form of nicotine dependence through e-cigarette use. While our study found statistically significant improvements in some sleep parameters, these changes were not clinically important. This highlights the complex nature of sleep quality and the multifaceted impact of both nicotine and tobacco on sleep architecture. Long-term studies are needed to fully understand how sustained use of nicotine-containing e-cigarettes might affect sleep quality over time.

## Conclusion

A smoking cessation intervention offering e-cigarettes for 6 months added to SOC counseling did not significantly alter participant’s quality of sleep and may even benefit individuals at higher risk of insomnia compared to SOC alone. Clinicians can inform patients willing to quit smoking with e-cigarettes that on average, e-cigarettes are unlikely to impact sleep outcomes. The observed group differences remained below the PSQI’s MCID.

## Supplementary Material

Scharf_ESTxENDS_Appendix(1)_zsag028

CONSORT_2025_20251111_zsag028

## Deposit of material in a data repository

The data will be accessible for analyses approved by the steering committee to avoid overlap with planned or ongoing analyses. Data sharing is possible starting 1 year after publication. Data can be requested via reto.auer@unibe.ch.
