# Supplementary material for: Effect of electronic nicotine delivery systems for smoking cessation on sleep quality: secondary analysis of a randomized controlled trial
Source: Sleep. 2026 Feb 5;49(7):zsag028. doi: 10.1093/sleep/zsag028 (PMC13357506; doi:10.1093/sleep/zsag028)
Supplement: Scharf_ESTxENDS_Appendix(1)_zsag028 [file scharf_estxends_appendix(1)_zsag028.docx]

Effect of electronic nicotine delivery systems for smoking cessation on sleep quality: secondary analysis of a randomized controlled trial

Tamara Scharf^1,2^, Anna Rihs^1^, Anna Schoeni^1^, Micheline Maire^3^, Kali Tal^1^, Julian Jakob^1,2,4^, Isabelle Jacot-Sadowski^5^, Jean-Paul Humair^6^, Aurélie Berthet^5^, Martin Brutsche^7^, Anja Frei^8^, Lucy Bolt^1,9^, Ramin Khatami^10^, Reto Auer^1,5^, Stéphanie Baggio^1,11^

^1^Institute of Primary Health Care (BIHAM), University of Bern, Switzerland, ^2^Graduate School of Health Sciences, University Bern, ^3^ Department of Internal Medicine, Stadtspital Zürich, Switzerland, ^4^Department of Paediatrics, University Hospital Bern, Inselspital, Bern, Switzerland, ^5^Center for Primary Care and Public Health (Unisanté), University of Lausanne, Switzerland, ^6^ Department of Primary Care Medicine, University Hospital of Geneva, Geneva, Switzerland, ^7^Lung Center, Kantonsspital St. Gallen, St. Gallen, Switzerland, ^8^University of Zurich, Epidemiology, Biostatistics and Prevention Institute, Zurich, Switzerland, ^9^Department of Internal Medicine, University Hospital Bern, Inselspital, Bern, Switzerland, ^10^Centre of Sleep Medicine and Epileptology Barmelweid, Klinik Barmelweid AG, Switzerland, ^11^Institute of Psychology, University of Lausanne, Switzerland

**Corresponding author**

Stéphanie Baggio

Institute of Primary Health Care (BIHAM)

University of Bern

Mittelstrasse 43

CH-3012 Bern

Switzerland

Email: stephanie.baggio@unibe.ch

Appendix

**Appendix Table S1**

**Unadjusted and adjusted models for sleep quality at 6 months comparing intervention and control groups for only baseline PSQI >5**

|  | **Unadjusted** | | **Final model with SI/IPAW**** | | |
| --- | --- | --- | --- | --- | --- |
| **Outcome** | **Slope (95% CI)** | **p-value** | **Slope (95% CI)** | **p-value** | **R^2^** |
| **PSQI score** | -0.93 (-1.64; -0.21) | **0.011** | -0.68 (-1.30; -0.06) | **0.031** | 0.30 |
| **Mean Change in PSQI score** | -0.56 (-1.24; 0.12) | 0.104 | -0.68 (-1.30; -0.06) | **0.031** | 0.21 |
| Sleep duration | 0.22 (-0.05; 0.50) | 0.107 | 0.31 (0.05; 0.58) | **0.021** | 0.13 |
| Sleep disturbance | -0.40 (-0.89; 0.09) | 0.112 | -0.41 (-0.99; 0.17) | 0.163 | 0.15* |
| Sleep latency | -0.97 (-6.44; 4.50) | 0.728 | -0.42 (-5.97; 5.14) | 0.883 | 0.11 |
| Dysfunction during the day | -0.48 (-0.91; -0.05) | **0.030** | -0.40 (-0.84; -0.06) | 0.085 | 0.08* |
| Sleep efficiency | 5.62 (2.68; 8.57) | **<0.001** | 5.10 (2.29; 7.91) | **<0.001** | 0.22 |
| Sleep quality | -0.25 (-0.66; 0.17) | 0.244 | -0.15 (-0.60; 0.31) | 0.531 | 0.09* |
| Medication for sleep | -0.22 (-0.69; 0.24) | 0.352 | -0.18 (-0.70; 0.34) | 0.502 | 0.10* |
| Change in sleep efficiency Baseline to Follow-up | 4.94 (1.61; 8.27) | **0.004** | 5.70 (2.44; 8.96) | **0.001** | 0.17 |

* Pseudo R^2^ are reported, as the outcomes were ordinal variables

** Multivariable adjusted model, adjusted for study site, age, gender, employment status, BMI, education, problematic substance use, use of medication influencing sleep, Patient Health Questionnaire-9 items score, Generalized Anxiety disorder-7 items score, baseline PSQI score, number of cigarettes per day, and Fagerström score, the model was stabilized with IPAW

Abbreviations: PSQI= Pittsburgh Sleep Quality Index; CI = Confidence Interval; SI = Simple Imputation; IPAW = Inversed Probability of Attrition Weights

**Appendix Figure S1: Mean Pittsburgh Sleep Quality Index (PSQI) score in participants with baseline PSQI >5**

**
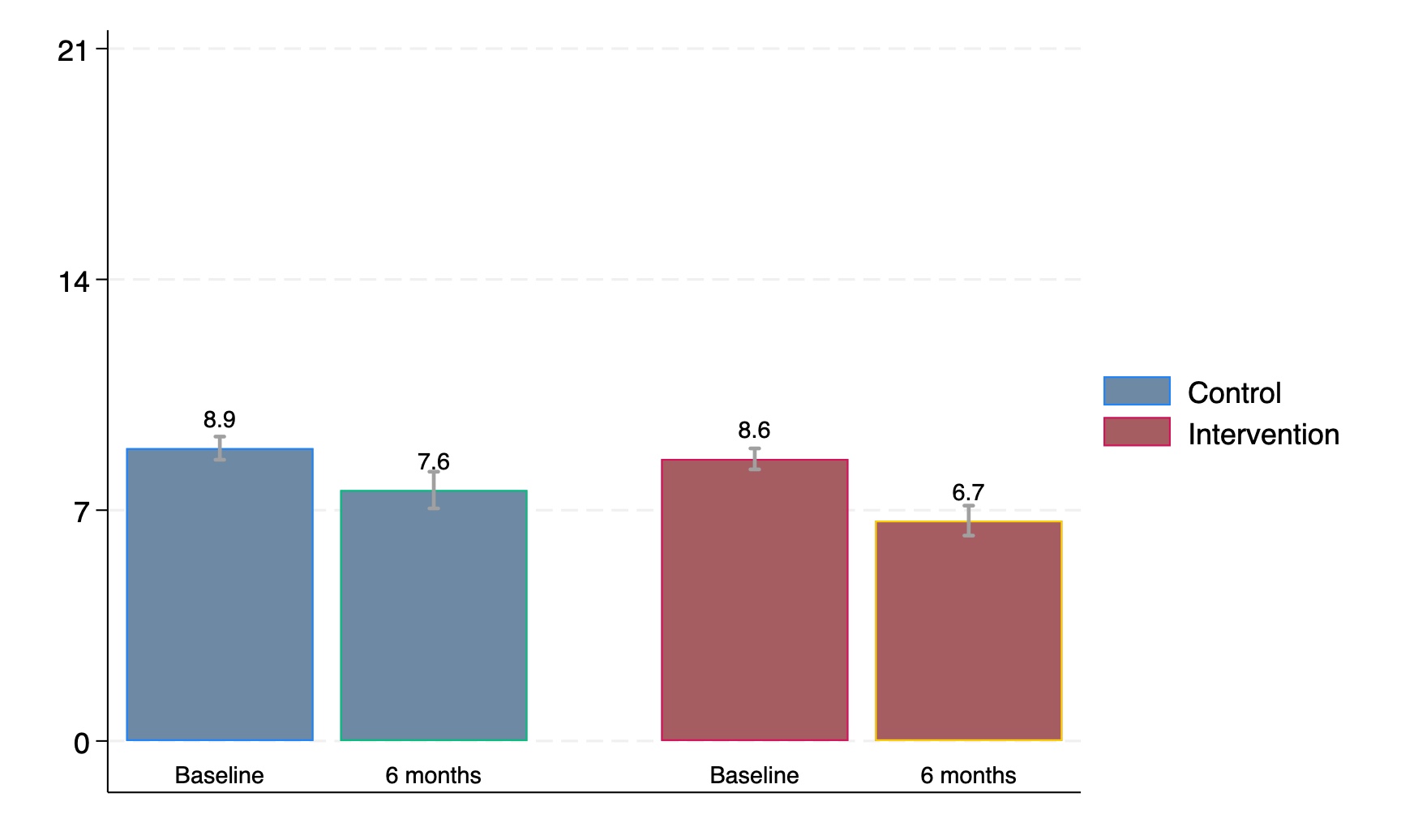
**

**Appendix Figure S2: Sleep efficiency for participants with baseline Pittsburgh Sleep Quality Index (PSQI) >5**


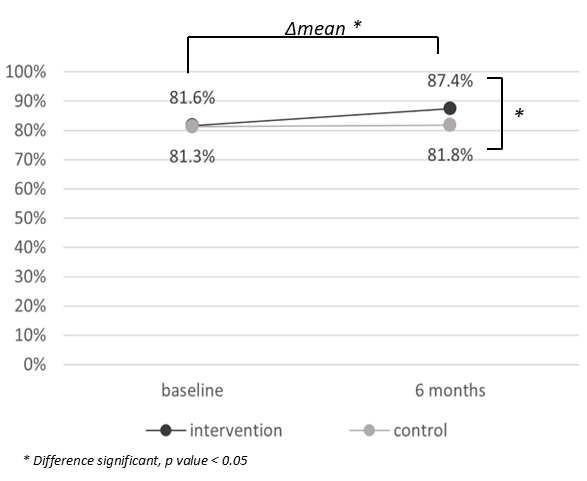


**Appendix Table S2.** **Characteristics of the participants in the intervention group at baseline grouped by 6-month tobacco/e-cigarette use**.

|  | Tobacco and e-cigarette abstainers | e-cigarettes with nicotine | e- cigarettes without nicotine | Cigarettes only | Dual users | Total |  |
| --- | --- | --- | --- | --- | --- | --- | --- |
|  | N=51 (11%) | N=193 (42%) | N=41 (9%) | N=91 (20%) | N=80 (18%) | N=456 |  |
| Age in years - median (IQR) | 35 (27-43) | 41 (31-54) | 40 (30-49) | 34 (26-45) | 36.5(28.5-54.5) | 38 (29-51) |  |
| Women gender - N (%) | 29 (56.9) | 88 (45.6) | 22 (53.7) | 41 (45.1) | 30 (37.5) | 210 (46.1) |  |
| Employed - N (%) | 40 (78.4) | 152 (78.8) | 31 (75.6) | 61 (67.0) | 55 (68.8) | 339 (74.3) |  |
| Highest educational qualification - N (%) |  |  |  |  |  |  |  |
| Obligatory school; other; none | 2 (3.9) | 14 (7.3) | 2 (4.9) | 4 (4.4) | 7 (8.8) | 29 (6.4) |  |
| Secondary education | 21 (41.2) | 93 (48.2) | 20 (48.8) | 43 (47.3) | 33 (41.3) | 210 (46.1) |  |
| Tertiary education | 28 (54.9) | 86 (44.6) | 19 (46.3) | 44 (48.4) | 40 (50.0) | 217 (47.6) |  |
|  |  |  |  |  |  |  |  |
| **Smoking behavior baseline** |  |  |  |  |  |  |  |
| Number of cigarettes per day – median (IQR) | | 15 (8-18) | 18 (12-20) | 15 (10-20) | 17 (10-20) | 15 (10-20) | 15 (10-20) |
| Participants with previous quit attempts (at least one) - N (%) | | 48 (94.1) | 166 (86.0) | 32 (78.1) | 74 (81.3) | 71 (88.8) | 395 (85.9) |
| Participants who smoke during the night - N (%) | | 5 (9.8) | 36 (18.7) | 6 (14.6) | 18 (19.8) | 19 (23.8) | 84 (18.4) |
| Fagerström Test for Nicotine Dependence - mean (SD) | | 3.0 (2.1) | 4.6 (2.1) | 4.1 (2.2) | 4.3 (2.3) | 4.4 (2.4) | 4.3 (2.3) |
|  | |  |  |  |  |  |  |
| **Sleep effecting characteristics** |  |  |  |  |  |  |  |
| Alcohol use - AUDIT-C score ≥ 3 women/≥4 men) - N (%) | 29 (56.9) | 114 (59.1) | 25 (61.0) | 57 (62.6) | 51 (63.8) | 276 (60.1) |  |
| Cannabis use at least once in lifetime - N (%) | 46 (90.2) | 158 (81.9) | 38 (92.7) | 82 (90.1) | 67 (83.8) | 391 (85.8) |  |
| PHQ-9 score - median (IQR) | 3 (1-6) | 3 (1-6) | 3 (2-5) | 3 (1-6) | 3 (1-6.5) | 3 (1-6) |  |
| GAD-7 score - median (IQR) | 5 (2-7) | 4 (2-7) | 3 (1-5) | 5 (2-7) | 4 (2-7) | 4 (2-7) |  |
| Participants with OSAS Diagnosis – N (%) | 0 | 8 (4.2) | 0 | 5 (5.5) | 6 (7.5) | 19 (4.1) |  |
| Mean PSQI score (SD) | 4.7 (2.5) | 5.5 (3.3) | 5.6 (3.1) | 4.9 (2.6) | 5.7 (3.4) | 5.3 (3.1) |  |
| PSQI >5 points – N (%) | 14 (27.5) | 76 (39.4) | 17 (41.5) | 32 (35.2) | 29 (36.3) | 168 (36.8) |  |

^a^ Scores range from 1 to 10, with higher scores indicating greater dependence.

Abbreviations: AUDIT-C: Alcohol Use Disorders Identification Test-Concise; GAD-7: Generalized Anxiety Disorder-7 items; IQR: Interquartile range; N: number or participants; PHQ-9: Patient Health Questionnaire-9 items; PSQI= Pittsburgh Sleep Quality Index; SD: standard deviation; OSAS: obstructive sleep apnea syndrome

**Appendix Table S3**

**Per-exposure analysis of the intervention group, comparing the sleep quality subscales at 6 months**

| **Groups** | **N (%)** | **Sleep efficiency** | | | **Sleep duration** | | | **Sleep onset latency** | | |
| --- | --- | --- | --- | --- | --- | --- | --- | --- | --- | --- |
|  |  | **Mean (SD)** | **Final model with SI/IPAW*** | | **Mean (SD)** | **Final model with SI/IPAW*** | | **Median (IQR)** | **Final model with SI/IPAW*** | |
|  |  |  | **Slope (95%CI)** | **p-value** |  | **Slope (95%CI)** | **p-value** |  | **Slope (95%CI)** | **p-value** |
| **Tobacco and e-cigarette abstainers** | 51 (11%) | 89.2 (8.3) | Ref. | Ref. | 6.9 (1.1) | Ref. | Ref. | 10 (5-30) | Ref. | Ref. |
| **e-cigarettes with nicotine only** | 193 (42%) | 89.0 (11.0) | 2.09  (-1.50; 5.67) | 0.254 | 6.7 (1.2) | 0.26  (-0.13; 0.63) | 0.188 | 15 (10-30) | 4.01  (-1.67; 9.69) | 0.166 |
| **e-cigarettes without nicotine only** | 41 (9%) | 87.6 (10.5) | 1.96  (-2.34; 6.26) | 0.371 | 6.8 (1.0) | -0.02  (-0.41; 0.38) | 0.937 | 12 (5-25) | -0.06  (-6.91; 7.03) | 0.987 |
| **Dual users** | 80 (18%) | 85.6 (13.4) | 2.77  (-1.46; 7.00) | 0.199 | 76.8 (1.3) | 0.11  (-0.32; 0.53) | 0.617 | 15 (10-30) | 5.10  (-1.55; 11.76) | 0.133 |
| **Exclusive smokers** | 91 (20%) | 89.6 (13.4) | -0.43  (-4.31; 3.46) | 0.830 | 6.8 (0.9) | 0.13  (-0.26; 0.52) | 0.515 | 15 (10-30) | 3.17  (-3.94; 10.27) | 0.382 |
|  |  | **R^2^ = 0.12** | | | **R^2^ = 0.16** | | | **R^2^ = 0.21** | | |

* Multivariable adjusted model, adjusted for study site, age, gender, employment status, body mass index, education, problematic substance use, use of medication influencing sleep, Patient Health Questionnaire-9 items score, Generalized Anxiety Disorder-7 items score, baseline Pittsburgh Sleep Quality Index score, number of cigarettes per day, and Fagerström score, the model was stabilized with IPAW

Abbreviations: CI: confidence interval; IPAW: inversed probability of attrition weights; Ref.: reference; SD: standard deviation; SI: simple imputation

**Appendix Table S4**

**Per-exposure analysis of the intervention group, comparing the sleep quality components at 6 months**

| **Groups** | **N (%)** | **Day disfunction** | | | **Sleep medication** | | | **Sleep quality** | | **Sleep disturbance** | |
| --- | --- | --- | --- | --- | --- | --- | --- | --- | --- | --- | --- |
|  |  | **Final model with SI/IPAW**** | | | **Final model with SI/IPAW**** | | | **Final model with SI/IPAW**** | | **Final model with SI/IPAW**** | |
|  |  | **Slope (95%CI)** | **p-value** | **Slope (95%CI)** | | **p-value** | **Slope (95%CI)** | | **p-value** | **Slope (95%CI)** | **p-value** |
| **Tobacco and e-cigarette abstainers** | 51 (11%) | Ref. | Ref. | Ref. | | Ref. | Ref. | | Ref. | Ref. | Ref. |
| **e-cigarettes with nicotine only** | 193 (42%) | 0.20  (-0.57; 0.97) | 0.613 | -0.47  (-1.39; 0.45) | | 0.321 | -0.48  (-1.09; -0.13) | | 0.122 | -1.50  (-2.45; -0.55) | **0.002** |
| **e-cigarettes without nicotine only** | 41 (9%) | 0.10  (-0.91; 1.00) | 0.848 | -0.01  (-1.20; 1.20) | | 0.995 | 0.37  (-0.52;1.25) | | 0.417 | -0.49  (-1.57; 0.59) | 0.370 |
| **Dual users** | 80 (18%) | -0.25  (-1.12; 0.62) | 0.571 | -0.29  (-1.37; 0.79) | | 0.602 | -0.53  (-1.23; 0.18) | | 0.142 | -0.96  (-2.06; 0.14) | 0.086 |
| **Exclusive smokers** | 91 (20%) | -0.09  (-0.87; 0.70) | 0.831 | -0.87  (-1.96; 0.21) | | 0.115 | -0.17  (-0.89; 0.56) | | 0.653 | -0.93  (-1.96; 0.10) | 0.076 |
|  |  | **R^2^ =0.13*** | | **R^2^ =0.17*** | | | **R^2^ =0.18*** | | | **R^2^ = 0.17*** | |

* = Pseudo R^2^ are reported, as the outcomes were ordinal variables

** Multivariable adjusted model, adjusted for study site, age, gender, employment status, body mass index, education, problematic substance use, use of medication influencing sleep, Patient Health Questionnaire-9 items score, Generalized Anxiety Disorder-7 items score, baseline Pittsburgh Sleep Quality Index score, number of cigarettes per day, and Fagerström score, the model was stabilized with IPAW

Abbreviations: CI: confidence interval; IPAW: inversed probability of attrition weights; Ref.: reference; SD: standard deviation; SI: simple imputation

**Appendix Table S5**

**Per-exposure analysis of the intervention group only comparing the sleep quality at 6 months for participants with baseline PSQI >5**

| **Groups** | **Mean PSQI score at 6 months**  **(SD)** | **Unadjusted** | | **Final model with SI/IPAW*** | | **Final model for mean PSQI change with SI/IPAW*** | |
| --- | --- | --- | --- | --- | --- | --- | --- |
|  |  | **Slope (95%CI)** | **p-value** | **Slope (95%CI)** | **p-value** | **Slope (95%CI)** | **p-value** |
|  |  |  |  | **R^2^ = 0.37** | | **R^2^ = 0.32** | |
| **Tobacco and e-cig. abstainers** | 7.0 (2.5) | Ref. | Ref. | Ref. | Ref. | Ref. | Ref. |
| **e-cig. with nicotine only** | 6.8 (3.1) | -0.18  (-1.95; 1.58) | 0.837 | 1.06  (-2.53; 0.40) | 0.153 | -1.06  (-2.53; 0.40) | 0.153 |
| **e-cig. without nicotine only** | 6.6 (2.5) | -0.41  (-2.60; 1.78) | 0.711 | -1.22  (-2.97; 0.54) | 0.172 | -1.22  (- 2.97; 0.53) | 0.172 |
| **Dual users** | 7.1 (3.6) | 0.07  (-1.90; 20.4) | 0.954 | -1.18  (-3.02; 0.66) | 0.207 | -1.18  (-3.02; 0.66) | 0.207 |
| **Exclusive smokers** | 6.0 (3.0) | -0.97  (-2.91; 0.97) | 0.326 | -1.02  (-2.77; 0.74) | 0.255 | -1.02  (-2.77; 0.74) | 0.255 |

* Multivariable adjusted model, adjusted for study site, age, gender, employment status, BMI, education, problematic substance use, use of medication influencing sleep, Patient Health Questionnaire-9 items score, Generalized Anxiety Disorder-7 items score, baseline PSQI score, number of cigarettes per day, and Fagerström score, the model was stabilized with inverse probability of attrition weights (IPAW)

Abbreviations: Cig.: cigarette; CI: confidence interval; IPAW: inversed probability of attrition weights; PSQI: Pittsburg Sleep Quality Index; Ref.: reference; SD: standard deviation; SI: simple imputation

**Appendix Table S6**

**Per-exposure analysis of the intervention group, comparing the sleep quality subscales at 6 months, stratified by 7 days point prevalence tobacco smoking abstinence (7-dppa) and sustained tobacco smoking abstinence***

| **Groups** |  | **Mean PSQI score at 6 months**  **(SD)** | **Unadjusted** | | **Final model with SI/IPAW*** | |
| --- | --- | --- | --- | --- | --- | --- |
|  | **N (%)** |  | **Slope (95%CI)** | **p-value** | **Slope (95%CI)** | **p-value** |
| **Sustained tobacco abstainers and e-cigarette abstainers** | 13 (3%) | 6.4 (4.6) | Ref. | Ref. | Ref. | Ref. |
| **Seven-day tobacco abstainers and e-cigarette abstainers** | 38 (8%) | 5.0 (3.1) | -1.41  (-3.32; 0.51) | 0.148 | -1.42  (-3.95; 1.11) | 0.269 |
| **e-cigarettes with nicotine** | 193 (42%) | 4.9 (2.9) | -1.53  (-3.24; 0.18) | 0.079 | -1.99  (-4.37;-0.38) | 0.099 |
| **e-cigarettes without nicotine** | 41 (9%) | 5.3 (2.9) | -1.07  (-2.97;0.83) | 0.270 | -1.48  (-3.97; 1.01) | 0.245 |
| **Dual users** | 80 (18%) | 5.3 (3.3) | -1.14  (-2.92; 0.65) | 0.212 | -1.60  (-4.08; 0.87) | 0.203 |
| **Exclusive smokers** | 91 (20%) | 4.7 (2.7) | -1.66  (-3.43; 0.11) | 0.066 | -1.80  (-4.21; 0.62) | 0.145 |
|  |  |  |  |  | **R^2^ =0.41** | |

* Multivariable adjusted model, adjusted for study site, age, gender, employment status, body mass index, education, problematic substance use, use of medication influencing sleep, Patient Health Questionnaire-9 items score, Generalized Anxiety Disorder-7 items score, baseline PSQI score, number of cigarettes per day, and Fagerström score, the model was stabilized with IPAW

Abbreviations: Cig.: cigarette; CI: confidence interval; IPAW: inversed probability of attrition weights; PSQI: Pittsburg Sleep Quality Index; Ref.: reference; SD: standard deviation; SI: simple imputation

**Appendix Table S7**

**Per-exposure analysis of the intervention group, comparing the sleep quality at 6 months, tobacco abstinence validated by urinary anabasine levels and if unavailable, by exhaled carbon monoxide (CO) levels***

| **Groups** |  | **Mean PSQI score at 6 months**  **(SD)** | **Unadjusted** | | **Final model with SI/IPAW**** | |
| --- | --- | --- | --- | --- | --- | --- |
|  | **N (%)** |  | **Slope (95%CI)** | **p-value** | **Slope (95%CI)** | **p-value** |
| **Validated tobacco abstainers and e-cigarette abstainers** | 44 (10%) | 5.5 (3.7) | Ref. | Ref. | Ref. | Ref. |
| **e-cigarettes with nicotine** | 193 (42%) | 4.9 (2.9) | -0.65  (-1.64; 0.35) | 0.204 | -1.19  (-2.15;-0.23) | **0.015** |
| **e-cigarettes without nicotine** | 41 (9%) | 5.3 (2.9) | -0.18  (-1.48;1.11) | 0.781 | -0.67  (-1.89; 0.54) | 0.278 |
| **Dual users** | 80 (18%) | 5.3 (3.3) | -0.25  (-1.37; 0.87) | 0.661 | -0.80  (-1.95; 0.35) | 0.170 |
| **Exclusive smokers or non-validated tobacco abstinence** | 98 (21%) | 4.7 (2.7) | -0.81  (-1.89; 0.28) | 0.144 | -1.05  (-2.10; 0.01) | 0.051 |
|  |  |  |  |  | **R^2^ =0.40** | |

* We defined biochemically validated abstinence with urinary anabasine levels (<3 ng/mL in urine), or if unavailable, exhaled carbon monoxide level of ≤9 ppm

** Multivariable adjusted model, adjusted for study site, age, gender, employment status, body mass index, education, problematic substance use, use of medication influencing sleep, patient Health Questionnaire-9 items score, Generalized Anxiety Disorder-7 items score, baseline Pittsburg Sleep Quality Index score, number of cigarettes per day, and Fagerström score, the model was stabilized with IPAW

Abbreviations: CI: confidence interval; IPAW: inversed probability of attrition weights; Ref.: reference; SD: standard deviation; SI: simple imputation
